# High level of unmet needs and anxiety are associated with delayed initiation of adjuvant chemotherapy for colorectal cancer patients

**DOI:** 10.1007/s00520-020-05333-z

**Published:** 2020-02-28

**Authors:** Li Zhu, Yi Xin Tong, Xiang Shang Xu, Ai Tang Xiao, Yu Jie Zhang, Sheng Zhang

**Affiliations:** Department of Gastrointestinal Surgery, Tongji Hospital, Tongji Medical College, Huazhong University of Science and Technology, Jie Fang Ave, No, Wuhan, 1095 China

**Keywords:** Colorectal cancer, Adjuvant chemotherapy, Delay initiation of chemotherapy, Patient’s needs, Anxiety, Depression, SCNS-SF34

## Abstract

**Aims:**

Adjuvant chemotherapy is recommended for patients with curatively resected colorectal cancer. The aim of this study is to evaluate the impact of unmet supportive care needs and anxiety on the initiation of postoperative adjuvant chemotherapy in colorectal cancer patients.

**Methods:**

This is a retrospective study from a single tertiary referral hospital. Patients diagnosed with colorectal cancer who met the inclusion criteria were included. The Hospital Anxiety and Depression Scale (HADS) and modified 34-item Supportive Care Needs Survey (SCNS-SF34) were applied to assess patient’s anxiety level and unmet needs. The time intervals between initiation of adjuvant chemotherapy and operation were recorded. Factors associated with delayed initiation of chemotherapy were investigated in univariate and multivariate analysis.

**Results:**

A total of 135 patients with colorectal cancer were included. In total, 16.3% (22/135) and 5.2% (7/135) reported symptoms of anxiety and depression. In multivariate analysis, low to moderate income status, postoperative complications, anxiety, and high level of unmet needs are independent risk factors for late initiation of chemotherapy.

**Conclusions:**

Our findings showed that psychological problems such as anxiety and high unmet supportive needs are correlated with delayed initiation of adjuvant chemotherapy in colorectal cancer patients.

**Electronic supplementary material:**

The online version of this article (10.1007/s00520-020-05333-z) contains supplementary material, which is available to authorized users.

## Introduction

Colorectal cancer (CRC) is the third most common cancer and the fourth leading cause of cancer death in the world [1]. Surgery together with adjuvant chemotherapy (AC) remains the standard treatment for stage III colorectal cancer patients and selected stage II patients [2–4]. The recently updated National Comprehensive Cancer Network (NCCN) guidelines suggested that the optimal timing to initiate postoperative adjuvant chemotherapies should be in/between 4 and 8 weeks after operations [5]. A systematic and meta-analysis involving 15,410 colorectal patients demonstrated that a 4-week increment in time to adjuvant chemotherapy was associated with a 14% decrease in both overall survival (OS) and disease-free survival (DFS) [6]. Another two large-scale retrospective studies from the US and Netherlands National Cancer Database also showed that a delay of 6–8 weeks between surgery and adjuvant therapy would reduce survival in stage II and III colorectal cancer patients [7, 8].

Multiple factors including patient demographics, advanced clinical stage, surgical techniques, and postoperative complications may correlate with the delay initiation of adjuvant chemotherapy [9, 10]. The lack of relevant knowledge may lead to patients’ distress and decease compliance to postoperative chemotherapy [11]. Many reports indicated that individualized cancer patients suffer from emotional disorders including anxiety and depression, which may compromise the effect of treatments [12]. Meanwhile, high level of unmet supportive care needs is prevalent among cancer survivors which may have a negative impact on clinical management [13].

The impact of patients’ psychological status and level of unmet needs on the start time of adjuvant chemotherapy has not been studied. This study focuses on exploring clinical and psychological factors associated with delayed initiation of adjuvant chemotherapy. We hypothesize that high level of anxiety and unmet supportive needs may lead to a delayed commencement of postoperative adjuvant chemotherapy in colorectal cancer patients.

## Method

### Study design and participants

In a single tertiary referral hospital, data from patients with colorectal cancer who underwent adjuvant chemotherapy between April 2017 and November 2018 were retrospectively collected and analyzed. Patients aged over 18 years who met the following inclusion criteria were recruited in the study: (1) histopathologically confirmed diagnosis of colorectal cancer (sites of the original were classified as colon and rectum); (2) are aware they will receive adjuvant chemotherapy and can communicate with our medical professional team. The exclusion criteria were (1) diagnosis of other malignancies; (2) patients already received neoadjuvant therapy; (3) plan to receive adjuvant chemotherapy in other hospitals; (4) with concurrent psychiatric disorder or other mental problems and fail to communicate with our team members. Ethical approval for the study was obtained from the institutional medical ethics committee. Informed consent was signed by all participants.

### Data collection

The following information was collected for analysis: (1) demographic characteristics such as age, gender, education status, socioeconomic status, patients’ performance status (according to classification of the Eastern Cooperative Oncology group) [14], and site of origin; (2) clinical or laboratory characteristics such as neutrophil to lymphocyte ratio (NLR), serum tumor markers as carcinoembryonic antigen (CEA) and carbohydrate antigen 19-9 (CA19-9), tumor size, differentiation, invasion depth (T), presence of lymph node metastases (N), presence of distant metastases (M), treatment, and surgical approach; (3) postoperative information such as postoperative complication (according to the Clavien-Dindo criteria [15]), length of stay, readmission, types of adjuvant chemotherapy, and initiation timing of chemotherapy.

### Anxiety/depression and supportive needs measurement

Hospital Anxiety and Depression Scale (HADS) was applied to assess self-reported symptoms of anxiety and depression. The HADS consists of 14 items: 7 for anxiety and 7 for depression. Participants answered the questions on a 4-point Likert scale. The total score for each scale ranges from 0 to 21. The cut-off value for definite cases of anxiety and depression is a score ≥ 11 [16, 17]. Supportive care needs for cancer patients were measured by the modified 34-item Supportive Care Needs Survey (SCNS-SF34). The modified version was adapted from the validated mandarin version of SCNS-SF34 with questions chosen from item 2–14, 17, 20–26, 28, and 29 [18]. We add 12 items specifically concerning relevant needs for chemotherapy to the modified version of SCNS-SF34. The modified SCNS-SF34 assesses cancer patients’ needs in five domains: physical and daily living (4 items), psychological (10 items), patient care and support (6 items), health systems and information (7 items), and chemotherapy (7 items) (Supplemental Table 1). Participants will indicate their level of need for the help for each item on a 5-point Likert scale: 1 = no need, not applicable; 2 = no need, satisfied; 3 = low need; 4 = moderate need; 5 = high need. A standardized Likert-summated score for each item ranging from 0 to 100 was calculated, with higher scores representing higher levels of need for help [18].

The Chinese version of HADS and modified SCNS-SF34 questionnaires were distributed to recruited participants in 48 h after administration. Two colorectal surgeons conducted the communication and explanation for patients with difficulties in understanding the items in each questionnaire. We collected all the questionnaires before discharge of the patients.

### Outcomes and follow-up

The primary endpoint of this study was the starting time of postoperative adjuvant chemotherapy. The secondary endpoint was the completion time and rate of adjuvant chemotherapy. We also evaluated the factors associated with delayed initiation of AC using multivariable analysis. We defined “early initiation” as the initiation of AC < 4 weeks and “delayed initiation” as the initiation of AC ≥ 8 weeks. The total follow-up period is 12 months. This study was approved by the ethics committees of Tongji hospital. The purpose and content of the study were explained to the participants. All participants signed the informed consent and confidentiality was assured.

### Statistical analyses

All continuous variables were presented as medians (range) and analyzed with the chi-square test or Mann-Whitney *U* test. Categorical variables were reported as whole numbers and percentages. The receiver operation curve (ROC curve) method was used to determine the cut-off value of modified SCNS-SF34 score as “low need” and “high need.” The Kaplan-Meier method was used to evaluate potential predictive factors for delayed initiation of adjuvant chemotherapy. Only factors with *p* value < 0.1 in univariate analysis were included in the final multivariate analysis model. Multivariate Cox regression was employed to identify independent predictive factors for delayed initiation of chemotherapy. All statistical tests were performed in the SPSS version 21 (IBM, Armonk, NY, USA) with a significance level of *p* value < 0.05.

## Results

### Patient baseline and clinicopathological characteristics

One hundred fifty-three patients were recruited and considered eligible for this study. After screening based on inclusion and exclusion criteria, 135 patients were successfully followed up and included in final study. The median age of included patients was 55.00 ± 9.73 and most patients were men (100/135, 74.1%). A total of 73.3% (99/135) patients were diagnosed with rectum cancer and 26.7% (36/135) were colon cancer. All patients underwent laparoscopic operation and 13.3% (18/135) of them were converted to open operation. In total, 4.4% (6/135) patients suffered from ≥ grade 3 postoperative complications and the median hospital stay is 16 ± 4.7 days. The median time between adjuvant chemotherapy and operation was 28 ± 14.9 days. The proportions of patients receiving AC within 4 weeks and 8 weeks were 59.3% (80/135) and 94.1% (127/135), respectively. A total of 73.3% (99/135) completed full cycles of adjuvant chemotherapy. Other information was summarized in Table 1.

**Table 1:** The demographic and clinicopathological characteristics of patient

Variable	Total (*n* = 135)
Baseline characteristics	
Age, median (IQR), year	55.00 ± 9.73
Gender	
Male Female	74.1% (100/135) 25.9% (35/135)
ECOG status	
0 1	8.1% (11/135) 91.9% (124/135)
Education	
Illiterate/primary school High school/undergraduate	26.7% (36/135) 73.3% (99/135)
Socioeconomic status	
Low to moderate income High income	78.5% (106/135) 21.5% (29/135)
Smoking	
Yes No	30.4% (41/135) 69.6% (94/135)
Alcohol	
Yes No	25.9% (35/135) 74.1% (100/135)
Clinicopathological parameters	
Location	
Colon Rectum	26.7% (36/135) 73.3% (99/135)
T stage (pT)	
T1 T2 T3 T4	0.7% (1/135) 15.6% (21/135) 55.6% (75/135) 28.1% (38/135)
N stage (pN)	
N0 N1 N2	55.6% (75/135) 29.6% (40/135) 14.8% (20/135)
Tumor size (IQR) cm	3.80 ± 1.30
Differentiation	
Well Medium Poor	9.6% (13/135) 63.7% (86/135) 26.7% (36/135)
Blood NLR (IQR)	2.41 ± 1.75
Serum CEA	
Normal Elevated	73.3% (99/135) 26.7% (36/135)
Serum CA19-9	
Normal Elevated	84.4% (114/135) 15.6% (21/135)
Operation	
Laparoscopic Conversion to laparotomy	86.7% (117/135) 13.3% (18/135)
Postoperative parameters	
Complication	
No Grades 1 and 2 ≥ Grade 3	86.7% (117/135) 8.9% (12/135) 4.4% (6/135)
Hospital stay, median, days	16 ± 4.7
Time between AC and operation, median, days	28 ± 14.9
AC within 4 weeks	
Yes No	59.3% (80/135) 40.7% (55/135)
AC within 8 weeks	
Yes No	94.1% (127/135) 5.9% (8/135)
AC regimen	
CapeOX SOX Capecitabine/S-1 FOLFOX6 Other	83.0% (112/135) 8.1% (11/135) 3.7% (5/135) 3.0% (4/135) 2.2% (3/135)
AC complete rate	
Yes No	73.3% (99/135) 26.7% (36/135)

*CI*, confidence interval; *IQR*, interquartile range; *ECOG*, Eastern Cooperative Oncology group; *CEA*, carcinoembryonic antigen; *CA19-9*, carbohydrate antigen; *NLR*, neutrophil to lymphocyte ratio; *AC*, adjuvant chemotherapy

### Prevalence of anxiety, depression, and supportive care needs

Among 135 colorectal cancer patients, 16.3% (22/135) and 5.2% (7/135) reported symptoms of anxiety and depression (HADS-anxiety or HADS-depression score ≥ 11) (Table 2). The average SCNS-SF34 standardized score of the patients was 54.2 ± 7.3. Half of the top 10 needs items were from the chemotherapy-related domain, two were from the patient care and support domain, and other three are of physical and daily living, psychological and health system, and information domain, respectively. The ten most frequently reported common unmet needs were listed in Table 3. Compared with patients with low unmet needs, patients with higher unmet needs showed significant lower percentage of early initiation of AC < 4 weeks (25.9% vs 81.5%) and higher percentage of delayed initiation of AC ≥ 8 weeks (14.8% vs 0%).

**Table 2:** The prevalence of anxiety and depression and supportive needs of patient

Variable	Total (*n* = 135)
Anxiety and depression	
HADS-anxiety score	
< 8 8–10 ≥ 11	61.5% (83/135) 22.2% (30/135) 16.3% (22/135)
HADS-depression score	
< 8 8–10 ≥ 11	72.6% (98/135) 22.2% (30/135) 5.2% (7/135)
SCNS-SF34 standardized score	54.2 ± 7.3
Low need (SCNS-SF34 score < 56) High need (SCNS-SF34 score ≥ 56)	60.0% (81/135) 40.0% (54/135)

*HADS*, Hospital Anxiety and Depression Scale; *SCNS-SF34*, 34-item Supportive Care Needs Survey

**Table 3:** Ten most frequently reported unmet needs (modified SCNS 34)

Rank	Modified SCNS-SF34 item	Domain	Average score	*N* (%) of unmet needs
1	Item 29, being informed the reason to receive chemotherapy after surgery and common side effects	Chemotherapy	73.6 ± 23.8	113/135 (85.7%)
2	Item 31, the common side effects of chemotherapy and how to handle them	Chemotherapy	73.4 ± 25.0	105/135 (77.8%)
3	Item 8, fears about the cancer spreading	Psychological	70.2 ± 25.6	104/135 (77.0%)
4	Item 28, being informed about the foods for recovery and foods to avoid	Chemotherapy	68.8 ± 23. 2	104/135 (77.0%)
5	Item 27, being informed about things you can do to help yourself to get well	Health system and information	68.2 ± 26.8	96/135 (71.1%)
6	Item 30, the time to start chemotherapy and how many cycles to receive	Chemotherapy	68.2 ± 27.4	96/135 (71.1%)
7	Item 20, cost of surgery and chemotherapy	Patient care and support	67.8 ± 27.2	98/135 (72.6%)
8	Item 18, financial support and insurance coverage for my disease and treatment	Patient care and support	66.6 ± 27.8	96/135 (71.1%)
9	Item 4, not being able to do the things you used to do	Physical and daily living	64.8 ± 26.6	95/135 (70.4%)
10	Item 32, emotional scared of chemotherapy	Chemotherapy	64.8 ± 27.8	94/135 (69.6%)

*SCNS-SF34*, 34-item Supportive Care Needs Survey

### Factors associated with the timing to adjuvant chemotherapy

Risk factors identified from univariate analysis were shown in Tables 4 and 5. Kaplan-Meier analysis showed that socioeconomic status, serum CEA, postoperative complications, HADS-anxiety status, HADS-depression status, and SCNS-SF34 score were significantly associated with early initiation (< 4 weeks) and delayed initiation (≥ 8 weeks) of adjuvant chemotherapy.

**Table 4:** Univariate and multivariate analysis for factors effecting early initiation (< 4 weeks) of AC

Variables	Univariate HR *p* value	Multivariate HR *p* value
Age		
≥ 75 < 75	1 (Ref) 1.176 0.625	NS
Socioeconomic status		
Low to moderate income High income	1 (Ref) 2.389 < 0.001	1 (Ref) 1.880 0.012
Education		
Illiterate/primary school High school/undergraduate	1 (Ref) 1.318 0.294	NS
Cancer TYPE		
Rectal Colon	1 (Ref) 1.332 0.236	NS
Serum CEA		
Elevated Normal	1 (Ref) 1.475 0.156	NS
Serum NLR		
≥ 2.4 < 2.4	1 (Ref) 1.366 0.221	NS
Complication		
Yes No	1 (Ref) 3.807 0.009	NS
HADS-anxiety		
Yes (≥ 11) No (< 11)	1 (Ref) 5.081 0.020	1 (Ref) 3.881 0.009
HADS-depression		
Yes (≥ 11) No (< 11)	1 (Ref) 5.866 0.079	NS
SCNS-SF34 score		
High need Low need	1 (Ref) 4.577 < 0.001	1 (Ref) 4.162 < 0.001

*HR*, hazard ratio; *CEA*, carcinoembryonic antigen; *AC*, adjuvant chemotherapy; *NLR*, neutrophil to lymphocyte ratio; *NS*, not significant

**Table 5:** Univariate and multivariate analysis for factors effecting delayed initiation (≥ 8 weeks) of AC

Variables	Univariate HR *p* value	Multivariate HR *p* value
Age		
< 75 ≥ 75	1 (Ref) 0.932 0.798	NS
Socioeconomic status		
High income Low to moderate income	1 (Ref) 1.979 0.002	1 (Ref) 2.159 0.001
Education		
High school/undergraduate Illiterate/primary school	1 (Ref) 1.136 0.526	NS
Cancer type		
Colon Rectal	1 (Ref) 1.014 0.944	NS
Serum CEA		
Normal Elevated	1 (Ref) 1.376 0.118	NS
Serum NLR		
< 2.4 ≥ 2.4	1 (Ref) 1.131 0.527	NS
Complication		
No Yes	1 (Ref) 2.451 0.002	1 (Ref) 2.263 0.006
HADS-anxiety		
No (< 11) Yes (≥ 11)	1 (Ref) 2.046 0.004	NS
HADS-depression		
No (< 11) Yes (≥ 11)	1 (Ref) 2.984 0.017	NS
SCNS-SF34 score		
Low need High need	1 (Ref) 3.182 < 0.001	1 (Ref) 2.905 < 0.001

*HR*, hazard ratio; *CEA*, carcinoembryonic antigen; *AC*, adjuvant chemotherapy; *NLR*, neutrophil to lymphocyte ratio; *NS*, not significant

In the multivariate analysis, socioeconomic status, postoperative complications, HADS-anxiety status, and high unmet supportive needs were identified as independent prognostic factors associated with early initiation and delayed initiation of adjuvant chemotherapy. For early initiation of chemotherapy, high income (*p* = 0.012), no anxiety (*p* = 0.009), and low unmet needs (*p* < 0.001) were significantly associated with early initiation of AC. For delayed chemotherapy ≥8 weeks, low to moderate income (HR = 2.159, *p* = 0.001), presence of postoperative complications (HR = 2.263, *p* = 0.006), and high unmet needs (HR = 2.905, *p* < 0.001) were independent risk factors. The details of multivariate analysis were listed in Tables 4 and 5.

## Conclusions

### Discussion of the results

A retrospective study was performed to evaluate clinical characteristics as well as psychological factors related to early and delayed initiation of adjuvant chemotherapy in colorectal cancer patients. Our results demonstrated that low unmet needs status is significantly correlated with early initiation of AC (HR = 4.162) while high unmet supportive needs is an independent risk factor for delayed initiation AC (HR = 2.905). In addition, high-income status (HR = 1.880) and no anxiety status (HR = 3.881) are correlated with early initiation of AC. A low to moderate income status (HR = 2.159) and presence of postoperative complication (HR = 2.263) are independent risk factors for delayed initiation of AC.

The initiation of chemotherapy treatment may have impact on patients’ psychological and physical states. Smooth transition to postoperative adjuvant chemotherapy is critical in colorectal cancer treatment. Although currently there is no consensus on the exact time to initiate adjuvant chemotherapy, it is generally agreed that the start time of chemotherapy should be no later than 8 weeks after operation [5–9]. The potential reasons for the delay of chemotherapy generally lie in the following aspects: patient-related factors such as lower socioeconomic status, female gender, and older age; treatment-related factors such as postoperative complications, surgical approach (open vs laparoscopic), and chemotherapy reagents; social and psychological factors such as lack of insurance, lack of social support, and insufficient knowledge on chemotherapy [10, 19–22]. In our study, we found that low income status and postoperative complications negatively correlated with the initiation of chemotherapy in colorectal cancer patients, which is in accordance with the present reports. In addition, we for the first time explored and demonstrated that anxiety status and high unmet needs may also correlate with the initiation time of chemotherapy.

Studies have also shown that most cancer patients experience certain level of emotional distress such as anxiety and depression [23]. It has been reported that the psychological problems such as anxiety and depression may lead to negative outcome in cancer perioperative treatment such as decreased adherence to oral chemotherapy [24]. Meanwhile, cancer patients suffered from various physical and psychological needs when they started chemotherapy [25]. Advanced stage, higher levels of distress, younger age, and woman gender are some predictors for higher level of supportive needs [26]. In our study, we found that colorectal cancer patients experience certain level of anxiety (16.3%) and depression (5.2%). Patients without anxiety tend to start AC within 4 weeks (HR = 3.881). It could be reasonable that patients suffered from anxiety may be afraid of postoperative chemotherapy and lead to the early delayed initiation. In addition, we also explored the prevalence of unmet supportive needs of colorectal cancer patients with modified SCNS-SF34 (simplified Chinese version) [18]. Our results supported our hypothesis that higher unmet needs status is a risk factor for delayed initiation of postoperative chemotherapy. Although previous studies showed that the perceived unmet needs of cancer patients are associated with poor psychological status and lower quality of life [27], our study for the first time showed that higher unmet needs postponed the initiation of postoperative chemotherapy.

It has been estimated that up to 50% of patients may experience a variety of physical and psychological disorders after diagnosis of cancer and during chemotherapy [28, 29]. Our results indicated that cancer patients experienced high level of unmet needs and may need support and practical help in the management of chemotherapy. Adjuvant chemotherapy is the standard treatment for stage III colorectal cancer patients after curative operation. There are few reports that have studied the techniques for improvement of chemotherapy adherence in patients with colorectal cancer. Furthermore, previous studies failed to prove the effectiveness of interventions in reducing the unmet needs in cancer patients [30]. In our study, there are a rather large proportion of patients with low education (26.7% illiterate/primary school) that might have certain bias in the questionnaires and might impede future educational intervention. Literatures reported that the proportion of low educational level (illiterate/primary school) of cancer patients in China varies from 20 to 50% [31–34], which is in accordance with our study. The educational level of cancer patients in China is generally in low level compared with that in developed countries. Therefore, personalized education and family member support program may be beneficial for colorectal cancer patients to a better management and adherence of adjuvant chemotherapy.

### Limitations

The present study has several limitations that should be taken into consideration. First, a relatively small number of patients were included in this study. Second, due to the retrospective setting, our study might inevitably have selection bias and some data might be missing. Third, we need further verification of the reliability of the modified SCNS-SF34 scale. In addition, information of outcome measurements, such as quality of life, patients’ satisfaction, and patient-reported adherence, were not evaluated in our study. Therefore, further perspective study with a larger sample size and more outcome measurements are needed to validate the finding of our study and further explore the effectiveness of individualized patient education program.

### Conclusion and clinical implications

Despite the limitations mentioned above, there are still many valuable implications of this retrospective study. First, to the best of our knowledge, our study for the first time indicated that high level of unmet needs and anxiety contributed to delayed initiation of adjuvant chemotherapy in patients with colorectal cancer. Second, our findings suggested that individualized education program to relieve patients’ anxiety and improve unmet needs might be effective in improving chemotherapy initiation and adherence. Based on our results and limitations, we hypothesized that cancer patients’ unmet need would have negative impact on chemotherapy adherence. Furthermore, we will further confirm the conclusion in a future well-designed randomized controlled trial and investigate various interventions to enhance patients’ adherence in chemotherapy.

## Electronic supplementary material

ESM 1 (DOCX 28 kb)

## References

[1] Siegel RL, Miller KD, Jemal A (2018). Cancer statistics, 2018. CA Cancer J Clin.

[2] Nelson H, Sargent DJ, Wieand HS, Fleshman J, Anvari M, The Clinical Outcomes of Surgical therapy Study Group (2004). A comparison of laparoscopically assisted and open colectomy for colon cancer. N Engl J Med.

[3] Hershman D, Hall MJ, Wang X, Jacobson JS, McBride R, Grann VR, Neugut AI (2006). Timing of adjuvant chemotherapy initiation after surgery for stage III colon cancer. Cancer.

[4] Benson AB, Schrag D, Somerfield MR, Cohen AM, Figueredo AT, Flynn PJ, Krzyzanowska MK, Maroun J, McAllister P, Van Cutsem E, Brouwers M, Charette M, Haller DG (2004). American Society of Clinical Oncology recommendations on adjuvant chemotherapy for stage II colon cancer. J Clin Oncol.

[5] National Comprehensive Cancer Network. Clinical practice guidelines in oncology. Colon Cancer Version 4.2018

[6] Biagi JJ, Raphael MJ, Mackillop WJ, Kong W, King WD, Booth CM (2011). Association between time to initiation of adjuvant chemotherapy and survival in colorectal cancer: a systematic review and meta-analysis. JAMA.

[7] Sun Z, Adam MA, Kim J, Nussbaum DP, Benrashid E, Mantyh CR, Migaly J (2016). Determining the optimal timing for initiation of adjuvant chemotherapy after resection for stage II and III colon cancer. Dis Colon rectum.

[8] Bos AC, van Erning FN, van Gestel YR, Creemers GJ, Punt CJ, van Oijen MG, Lemmens VE (2015). Timing of adjuvant chemotherapy and its relation to survival among patients with stage III colon cancer. Eur J Cancer.

[9] Czaykowski PM, Gill S, Kennecke HF, Gordon VL, Turener D (2011). Adjuvant chemotherapy for stage III colon cancer: does timing matter?. Dis Colon rectum.

[10] Malietzis G, Mughal A, Currie AC, Anyamene N, Kennedy RH, Athanasiou T, Jenkins JT (2015). Factors implicated for delay of adjuvant chemotherapy in colorectal cancer: a meta-analysis of observational studies. Ann Surg Oncol.

[11] Farrell, C, Brearley, S.G, Pilling, M, Molassiotis, A (2013) The impact of chemotherapy-related nausea on patients’ nutritional status, psychological distress and quality of life. Support Care Cancer 21(1):59–66 10.1007/s00520-012-1493-9 22610269

[12] Krebber AM, Buffart LM, Kleijn G, Riepma IC, Bree R, Leemans CR (2014). Prevalence of depression in cancer patients: a meta-analysis of diagnostic interviews and self-report instruments. Psycho Oncol.

[13] Armes J, Crowe M, Colbourne L, Morgan H, Murrells T, Oakley C, Palmer N, Ream E, Young A, Richardson A (2009). Patients’ supportive care needs beyond the end of cancer treatment: a prospective, longitudinal survey. J Clin Oncol.

[14] Oken MM, Creech RH, Tormey DC, Horton J, Davis TE, McFadden ET, Carbone PP (1982). Toxicity and response criteria of the Eastern Cooperative Oncology Group. Am J Clin Oncol.

[15] Clavien PA, Barkun J, de Oliveira ML, Vauthey JN, Dindo D, Schulick RD, de Santibañes E, Pekolj J, Slankamenac K, Bassi C, Graf R, Vonlanthen R, Padbury R, Cameron JL, Makuuchi M (2009). The Clavien-Dindo classification of surgical complications: five-year experience. Ann Surg.

[16] Zigmond AS, Snaith RP (1983). The hospital anxiety and depression scale. Acta Psychiatr Scand.

[17] Ye WF, Xu JM (1993). An evaluation of the hospital anxiety and depression scale in the general hospital patients. China Acts of Medical Sciences.

[18] Han Y, Zhou Y, Wang J, Zhao Q, Qin H, Fan Y, Song Y, Boyes A, Cui S (2017). Psychometric testing of the Mandarin version of the 34-item Short-Form Supportive Care Needs Survey in patients with cancer in mainland China. Support Care Cancer.

[19] Buyse M, Zeleniuch-Jacquotte A, Chalmers TC (1988). Adjuvant therapy of colorectal cancer. Why we still don’t know. JAMA.

[20] Kim YW, Choi EH, Kim BR, Ko WA, Do YM, Kim IY (2017). The impact of delayed commencement of adjuvant chemotherapy (eight or more weeks) on survival in stage II and III colon cancer: a national population-based cohort study. Oncotarget.

[21] Bayraktar S, Bayraktar UD, Rocha-Lima CM (2010). Timing of adjuvant and neoadjuvant therapy in colorectal cancers. Clin Colorectal Cancer.

[22] Park HS, Jung M, Kim HS, Kim HI, An JY, Cheong JH, Hyung WJ, Noh SH, Kim YI, Chung HC, Rha SY (2015). Proper timing of adjuvant chemotherapy affects survival in patients with stage 2 and 3 gastric cancer. Ann Surg Oncol.

[23] Zabora J, BrintzenhofeSzoc K, Curbow B, Hooker C, Piantadosi S (2001). The prevalence of psychological distress by cancer site. Psychooncology.

[24] Font R, Espinas JA, Layos L, Martinez Villacampa M, Capdevila J, Tobena M (2017). Adherence to capecitabine in preoperative treatment of stage II and III rectal cancer: do we need to worry?. Ann Oncol.

[25] Garcia MA, Kalecinski J, Oriol M, Bonne A, Lofti M, Espenel S, Tinquaut F, Collard O, Vassal C, Rivoirard R, Regnier V, Chauvin F, Bourmaud A (2018). Cancer patients treated with intravenous chemotherapy for the first time. What are their needs? What do they lack?. A qualitative–quantitative mixed approach Patient Prefer Adherence.

[26] Fiszer C, Dolbeault S, Sultan S, Brédart A (2014). Prevalence, intensity, and predictors of the supportive care needs of women diagnosed with breast cancer: a systematic review. Psychooncology.

[27] Akechi T, Okuyama T, Endo C (2011). Patient’s perceived need and psychological distress and/or quality of life in ambulatory breast cancer patients in Japan. Psychooncology.

[28] Parle M, Jones B, Maguire P (1996). Maladaptive coping and affective disorders among cancer patients. Psychol Med.

[29] Ford S, Lewis S, Fallowfield L (1995). Psychological morbidity in newly referred patients with cancer. J Psychosom Res.

[30] Girgis A, Breen S, Stacey F, Lecathelinais C (2009). Impact of two supportive care interventions on anxiety, depression, quality of life, and unmet needs in patients with nonlocalized breast and colorectal cancers. J Clin Oncol.

[31] Xie FL, Wang YQ, Peng LF, Lin FY, He YL, Jiang ZQ (2017). Beneficial effect of educational and nutritional intervention on the nutritional status and compliance of gastric cancer patients undergoing chemotherapy: a randomized trial. Nutr Cancer.

[32] Li Z, Wei D, Zhu C, Zhang Q (2019). Effect of a patient education and rehabilitation program on anxiety, depression and quality of life in muscle invasive bladder cancer patients treated with adjuvant chemotherapy. Medicine (Baltimore).

[33] Yanwei L, Minghui F, Manman Q, Zhuchun Y, Dongying L, Zhanyu P (2018). Influence of wellness education on first-line icotinib hydrochloride patients with stage IV non–small cell lung cancer and their family caregivers. Curr Probl Cancer.

[34] Zhang M, Chan SW, You L, Wen Y, Peng L, Liu W, Zheng M (2014). The effectiveness of a self-efficacy-enhancing intervention for Chinese patients with colorectal cancer: a randomized controlled trial with 6-month follow up. Int J Nurs Stud.

