# Supplementary material for: High level of unmet needs and anxiety are associated with delayed initiation of adjuvant chemotherapy for colorectal cancer patients
Source: Support Care Cancer. 2020 Feb 28;28(11):5299–306. doi: 10.1007/s00520-020-05333-z (PMC7547036; doi:10.1007/s00520-020-05333-z)
Supplement: Supplementary file 1 — (DOCX 28 kb) [file 520_2020_5333_MOESM1_ESM.docx]

Supplemental Table 1. Modified SCNS-SF34-C (Mandarin) form

| Domain of needs | Items |
| --- | --- |
| Physical and daily living (4 items) | 1. Lack of energy/tiredness  2. Feeling unwell a lot of the time  3. Work around the home  4. Not being able to do the things you used to do |
| Psychological  (10 items) | 5. Axiety  6. Feeling down and depressed  7. Feeling of sadness  8. Fears about the cancer spreading  9. Worry that the results of surgery or chemotherapy are beyond control  10. Uncertainty about the future  11. Learning to feel in control of your situation  12. Keeping a positive outlook  13. Feelings about death and dying  14. Concerns about the worries of those close to you |
| Patient care and support (6 items) | 15. Reassurance by medical staff that the way you feel is normal  16. Hospital staff attending promptly to your physical and emotional needs  **17. Supports from family members and friends**  **18. Financial support and insurance coverage for my disease and treatment**  **19. Personal or emergency contact for follow-up examination and re-adiministration**  **20. Cost of surgery and chemotherapy** |
| Health system and information (7 items) | 21. Being given written information about the important aspects of your care  22. Being given information (written, diagrams, drawings) about aspects of managing your illness and side-effects at home  23. Being given explanations of those tests for which you would like explanations  24. Being adequately informed about the benefits and side-effects of treatments before you choose to have them  **25. Being informed about educational programs that provided by medical professionals**  26. Being informed about cancer which is under control or diminishing (that is, in remission)  27. Being informed about things you can do to help yourself to get well |
| Chemotherapy  (7 items) | **28. Being informed about the foods for recovery and foods to avoid**  **29. Being informed the reason to receive chemotherapy after surgery and common side-effects.**  **30. The time to start chemotherapy and how many cycles to receive.**  **31. The common side-effects of chemotherapy and how to handle them**  **32. Emotional scared of chemotherapy**  **33. Physical and appearance concerns during chemotherapy**  **34. The plan for follow-up examination during and after chemotherapy** |

* The items in bold indicate the added 12 items of cancer patient needs specifically concerning chemotherapy
